# Seat Choice in a Crowded Café: Effects of Eye Contact, Distance, and Anchoring

**DOI:** 10.3389/fpsyg.2019.00331

**Published:** 2019-02-19

**Authors:** Henk Staats, Piet Groot

**Affiliations:** ^1^Department of Social and Organisational Psychology, Leiden University, Leiden, Netherlands; ^2^Department of Social, Health and Organizational Psychology, Utrecht University, Utrecht, Netherlands

**Keywords:** privacy, intimacy, affiliative conflict theory, seat choice, café, pleasure, arousal

## Abstract

According to theories of interpersonal distance people choose to position themselves in relation to nearby others in a way that optimizes intimacy and privacy. In two studies we investigated the influence of intimacy and privacy on seating behavior in a café (coffee house) setting. In Study 1 (*N* = 71) we manipulated two aspects of intimacy (eye contact and distance to others), and one aspect of privacy (architectural anchoring) in separate scenario’s and registered participants’ seat choice on floor plans of the three hypothetical cafés. We found that more often participants chose a seat that was at a larger distance to other café-goers. Study 2 (*N* = 121) replicated the design of the first study, but included affective and cognitive appraisal measures concerning both available seats in each scenario. This time we found that participants more often chose low-eye contact and anchored seats. Choices in line with hypotheses as well as those that were against hypotheses co-occurred with strong beliefs about the pleasure and arousal that each choice might provide and related to the expectations of interaction with others present. Results qualify expectations about protection and violation of intimacy and privacy, at least for café settings.

## Introduction

Imagine going to a café when you are alone in town. You’re craving a cup of coffee, but as you enter the place you notice that most seats have already been taken by other customers. In fact, there are only two options left—one seat at a small table near the corner, and another seat at a larger table near the bar. Taking either seat would imply that you would have to share the table with others. You really want that coffee, so leaving is not an option. Which of the two tables would you choose to sit at?

Anyone who frequents coffee houses may be familiar with the above scenario. In modern cities, coffee houses or ‘cafés,’ as they are sometimes called, can be found in abundance. They can serve as a pleasant ‘getaway’ from daily routine, and people go there not just to get their coffee, but also to meet up with friends, relax, or engage in discussions with strangers (Oldenburg and Brissett, 1982). For café owners, it is important to know to which kind of seats customers respond positively and to which they don’t, to run a café that invites people to stay.

In this article, we investigate the impact of three manipulations of the interior design of a café on seat choice. More specifically, we report two studies in which we examine how an individual café-goer’s seat choice is affected by the possibility of eye contact with a nearby person, by physical distance to others in the café, and by the degree of physical shielding from other persons, manipulations of intimacy and privacy that we derived from affiliative conflict theory (Argyle and Dean, 1965) and privacy regulation theory (Altman, 1975). In each of the two studies, participants responded to floor plans of a café interior in three separate scenarios, in which either intimacy or privacy levels were manipulated, and indicated which one of two available seats they preferred. With that we aimed to obtain proof of the tenability and replicability of hypotheses based on the intimacy and privacy premises in this particular setting. In the second study we additionally included affective and cognitive appraisals of both the chosen and the non-chosen seat, to provide a deeper understanding of the choice process as a function of intimacy or privacy concerns. In the next paragraphs we briefly review the literature regarding interpersonal distance, intimacy and privacy to derive hypotheses about spatial choice behavior in a café setting.

### Interpersonal Distance

How a person behaves in a given environment depends both on the presence of other people in that environment and on the qualities of the environment itself. Hall (1968) noted that in Western society there are four regions of interpersonal distance in which interaction takes place, which he labeled the intimate domain (0–45 cm distance between persons), personal domain (45–120 cm), social-consultative domain (120–400 cm.), and public domain (400+ cm). Depending on the quality of the relationship between people, they will feel comfortable interacting in any of these four domains. Intrusions of personal space—when another person enters an intimate space domain without permission—can cause discomfort to the person whose space was invaded. For example, when in an early study the personal space of an unsuspecting participant was invaded by the experimenter, the victim responded by facing away and eventually by fleeing the uncomfortable scene (Felipe and Sommer, 1966). The same kind of behavior was later observed in a library, where people avoided seats that were within each other’s personal domain (Eastman and Harper, 1971), and in a shopping mall, where people kept a larger distance to strangers than to familiars (Burgess, 1983). Besides having one’s own space domain invaded by someone else, it can also be uncomfortable to invade other people’s space. When participants were instructed to walk through the personal domain of two conversing people, the participants reported bad mood and showed avoidant non-verbal behavior (Efran and Cheyne, 1974). Even in virtual space people’s behavior is guided by concerns of intimacy and privacy. For example, Han et al. (2015) found that feelings of privacy and of others’ responsiveness promoted a sense of social presence among Twitter users, which in turn promoted the feeling of gratification of social connection with others. In other virtual environments researchers have found support for the existence of personal space between virtual avatars (Nassiri et al., 2010) and for participants immersed in virtual reality (Iachini et al., 2016). In both latter studies, participants maintained their distance to a virtual person, similar to real life. Finally, personal distance and gaze direction have also been identified as important cues for interaction between humans and robots, in the situation where a human customer orders a drink from a robot bartender (Loth et al., 2015).

Two influential theories of personal distance focus on intimacy and privacy to explain people’s behavior in response to nearby others: Affiliative conflict theory addresses people’s need for intimacy and distance (Argyle and Dean, 1965), and privacy regulation theory addresses people’s need for privacy defined as “selective control of access to the self or to one’s group” (Altman, 1975, p. 18). By using these two theories, we explain how spatial and social factors can influence seat choice of a café-goer.

### Intimacy and Privacy

Affiliative conflict theory (Argyle and Dean, 1965; see Patterson, 1973) states that individuals when interacting with other people desire to strike a balance between two contrasting needs: the need to achieve intimacy and the need to maintain individuality and freedom. This equilibrium can be realized in multiple ways. For example, one can alter one’s physical distance to another person, the amount of eye contact with the other, or the conversation topic; deviations from the intimacy equilibrium on one component (e.g., physical proximity to the other person) can be compensated by altering another (e.g., eye contact). Failing to compensate can result in distress: In case of too much intimacy, individuals will feel anxious about revealing inner states and about being rejected; and in case of too little intimacy, individuals will feel lonely, which also leads to distress (Argyle and Dean, 1965). Supporting this notion, Middlemist et al. (1976) found that people got aroused when their personal space was invaded in a place where compensation was impossible (i.e., the lavatory). The relationship between intimacy and arousal was further demonstrated by Coutts et al. (1980), who found that a sudden, substantial increase in intimacy of an accomplice led to an increase in the subject’s arousal.

Somewhat similar to affiliative conflict theory, privacy regulation theory (Altman, 1975) predicts that individuals will feel most satisfied when there is a match between their desired and achieved amount of privacy, which Altman (1975, p. 18) defined as “selective control of access to the self or to one’s group.” He goes on to describe that, when the amount of privacy that is desired by the individual exceeds the actual amount of privacy that the individual can achieve (i.e., there is not enough privacy), an individual will experience crowding. Conversely, when the amount of actual privacy exceeds the amount of desired privacy (i.e., there is more privacy than desired by the individual) an individual will experience social isolation or loneliness^1^ (Altman, 1975).

Although the concept of privacy seems similar to the intimacy equilibrium proposed by the affiliative conflict model (Argyle and Dean, 1965), it is possible to formulate some conceptual differences between the two constructs. First of all, intimacy, as conceptualized by Argyle and Dean (1965), is described as the result of approach and avoidance tendencies between two interacting individuals. This intimacy is said to be a function of eye contact, physical proximity, intimacy of conversation topic, amount of smiling, etc., and is thought to result from, among other things, the need for social feedback (Argyle and Dean, 1965). Privacy, on the other hand, is described by Altman (1975, p. 18) as “selective control of access to the self or to one’s group,” and so privacy regulation theory emphasizes the importance of control over access to the self. We therefore argue that affiliative conflict theory speaks of intimacy as a—more-or-less two-sided—phenomenon resulting from the interaction between two individuals; and that privacy regulation theory speaks of privacy as a—more-or-less one-sided—phenomenon resulting from control over access to the self. In other words, let intimacy be defined as psychological closeness resulting from interpersonal behavior, in situations where both persons are defined by the interaction; and let privacy be defined as selective control of access to the self or to one’s group, in situations where the observer may remain undefined. In our view this difference between the theories has not been emphasized before, and allows the study of different situations within a specific setting. This creates the possibility to compare the strength of potential defense strategies and the affective and cognitive appraisals that originate from these different situations.

To summarize, affiliative conflict theory and privacy regulation theory explain that humans regulate their interpersonal behavior in order to maintain optimal levels of intimacy and privacy. However, the theories make different predictions about the mechanisms through which this regulation takes place and also deal with slightly different situations: Affiliative conflict theory deals with interpersonal settings wherein the regulation mechanism is intimacy between persons who are present; privacy regulation theory designates privacy as the regulation mechanism of social input and output, and focuses on the individual as a recipient of social stimuli transferred by others who may be undefined, are not present yet but only anticipated (Baum and Greenberg, 1975; Holland et al., 2004) or who are present, i.e., in the same environment but not in any way interacting with the person (Robson, 2002). It is not clear how these privacy and intimacy mechanisms will operate in the café setting. Individuals’ intimacy and privacy preferences are not constant over time (Aiello, 1987), and depend on properties of the environment—e.g., the density of people (e.g., Fuhrer, 1987); the stressfulness of the occasion (Robson, 2008); the situational norm (Aarts and Dijksterhuis, 2003)—and on properties of the person, such as gender (e.g., Yildirim and Akalin-Baskaya, 2007) and personality (Aiello et al., 1977; Gifford and Gallagher, 1985). In other words, the intimacy and privacy equilibria do not lead to clear behavioral standards, as these standards may be moderated by individual and situational characteristics. This raises the question how intimacy and privacy influence social behavior in cafés, since cafés are places where social contact among strangers is a common phenomenon and indeed perhaps the norm (Oldenburg and Brissett, 1982).

### Overview of the Experiments and Hypotheses

In order to investigate seating choice of café-goers, in two studies we used intimacy and privacy theory to manipulate the attractiveness of different available seats. Our general aim was to investigate whether the traditional hypotheses, predicting a tendency to protect against intimacy regarding strangers, and to keep up privacy by maximizing control over interactions, will hold in a setting that is generally considered to have an important social function. In Study One, two scenarios were derived from affiliative conflict theory and one from privacy regulation theory. In the two affiliative conflict scenarios, intimacy was manipulated by changing the amount of eye contact to persons and the degree of physical distance with persons seated at the same table. A typical square table of up to four seats, as often used in a café, has sides that range in length from 70 to 100 cm (28–40 inches; Sommer, 1965; IKEA online catalogue, 2014), which is within a person’s personal distance zone (Hall, 1968).

Argyle and Dean (1965) found that individuals who were uncomfortably close to one-another compensated by reducing their eye contact. Therefore, we predicted that participants would prefer a seat that reduced eye contact when that seat was located at a café table with another person present (Hypothesis 1a).

**FIGURE 1:**
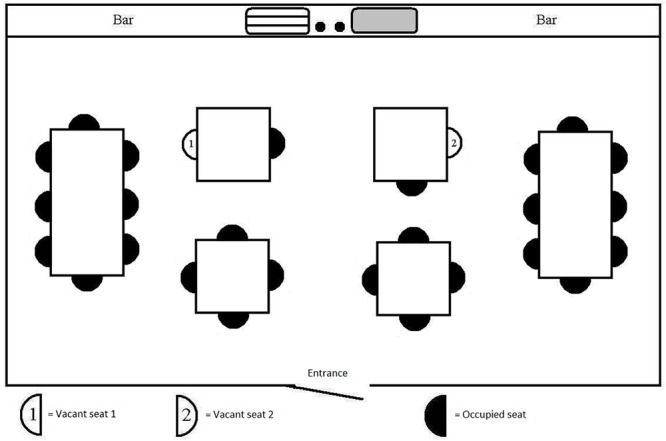
Manipulation of eye contact.

We also predicted that when individuals could choose between a single-length and a double-length table of which all but one seats were taken, they would prefer the double-length table to optimize inter-personal distance (Hypothesis 1b). These two hypotheses were tested in scenario 1 and 2 (see Figure 1A,B).

In the privacy regulation scenario (Figure 1C), privacy was manipulated by altering the amount of possible input regulation by ‘anchoring’ one of two tables to a wall (see Robson, 2008). This shielded the vacant seat from café-goers seated at other tables, but not from those that were seated at the same table. This way, privacy was manipulated while keeping intimacy levels constant. Robson (2008) found that individuals preferred seats that were ‘anchored’ in the environment in order to increase their privacy. We argued that lone café-goers would prefer a seat that facilitated social input regulation, which was a seat at a table anchored to a wall, over a non-anchored table (Hypothesis 1c).

In Study 2 we used the same scenarios as in the first study but added measures of affect and cognition to our dependent variables in order to gain a deeper understanding of the motivational forces underlying intimacy and privacy. Specifically, we measured affective responses and social cognitions regarding the vacant seats to uncover the patterns that would emerge for the intimacy and the privacy scenarios. Two hypotheses were derived from the circumplex model of affect (Russell, 1980), which states that humans will show an affective response to the environment that can be mapped onto the two orthogonal dimensions ‘pleasure’ and ‘arousal.’ We predicted that seat choice would be positively related to pleasure ratings of the chosen seat (Hypothesis 2a). Furthermore, given the association between unwanted intimacy and arousal previously demonstrated (e.g., Middlemist et al., 1976; Coutts et al., 1980; Evans and Wener, 2007) we predicted that seat choice would be negatively related to arousal ratings of the chosen seat (Hypothesis 2b). In Study 2 we also measured cognitive responses to the available seats (taken from Staats and Van der Jagt, unpublished). We aimed to explore whether cognitive and affective responses could explain seat choice. Participants not only rated the seat they chose, on the given affective and cognitive attributes, but they also rated the non-chosen seat on the same attributes, thus providing information about the relative quality of each seat. We anticipated that choice might be positively motivated by the attractiveness of one of the two options, but could also be negatively motivated to avoid the unattractive option. This comparative approach can uncover the reasons for such a choice.

## Study 1

In three different scenarios, participants were asked to indicate on a floor plan of a café which seat from two available seats they preferred, while the café was otherwise completely occupied with people unacquainted with the participant. Each empty seat stood at a table at which all other seats were already occupied.

### Methods

#### Participants, Consent Procedure, and Design

Participants were 71 pre-university students attending an introductory psychology lecture at a university in the Netherlands (86% female, *M*_age_ = 17.3 years, *SD* = 0.96). As part of the lecture, students were asked to participate in the current experiment. They were advised that participation was voluntary, and could be discontinued at any time they wished without penalty, and that their data would be anonymized. Verbal informed consent was obtained for all participants. A prospective ethics approval was not required as per the Institution’s guidelines and national regulations at the time the research was conducted. The study has been retrospectively approved by the Ethics committee of the Leiden University Institute of Psychology on September 6, 2018, while preparing this paper. All participants received the same questionnaire, which contained the three different café scenarios that were presented in a fixed order.

#### Manipulations

##### The café scenarios

Participants were instructed to imagine that they were going to a café. Each scenario was preceded by the following description of the café atmosphere (translated from Dutch)*: “You are going to a café downtown. In this café there are many people, and, besides two empty seats, the café is full. In the background music is playing softly. You will be spending 1 h in this café.”*

##### Seating arrangements

Each scenario was accompanied by the floor plan of the imaginary café. The floor plans served to visualize the café scenario, for it has been found that floor plans can effectively communicate a comprehensible mental image of an environment to a target person (Al-Kodmany, 2002).

**FIGURE 2:**
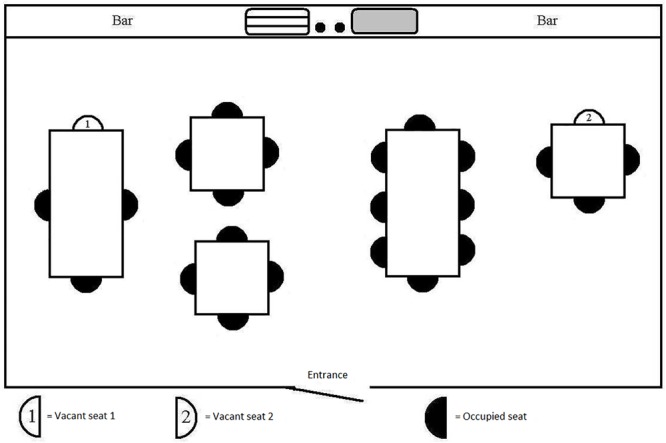
Manipulation of distance.

**FIGURE 3:**
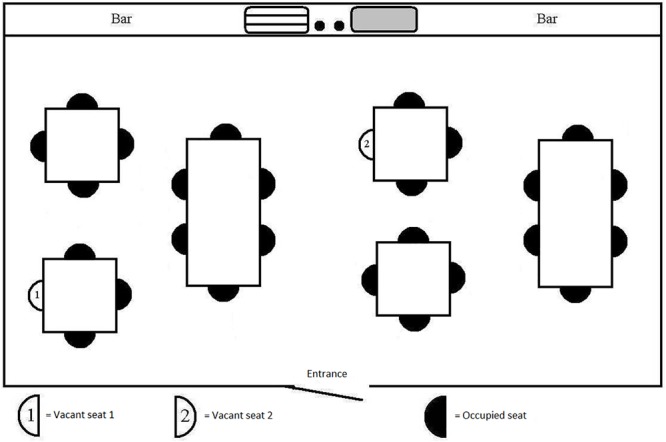
Manipulation of anchoring.

The floor plans were of a very simple design. The café interior was rectangular in shape; it contained no other elements than an entry door, several tables and seats, and a bar. Of the 24 (in the *distance* scenario) to 28 seats (in the *eye contact* and *anchoring* scenarios) that were depicted, each time only two seats were still vacant in the café; these were labeled *1* and *2*, and were located at two separate tables. All other seats were colored black and had no number, and the legend stated that these were occupied (see Figure 1–3). The vacant seat was always part of a table setting that had other seats that were all taken: thus the participant had to choose between two tables that both seated other café-goers. Within each separate scenario the number of taken seats at the table was held equal for both tables.

In each of the three scenarios that were tested, the interior of the café was slightly different. For the *“eye contact”* scenario (Figure 1), the angle at which the participant would be seated to one other café-goer at the same table was varied. This angle was either 180° (the participant would be seated directly opposite to another café-goer) or 90° (the participant would be seated perpendicularly to the other café-goer).

For the *“distance”* scenario (Figure 2), distance between the participant and three other café-goers seated at the same table was varied between tables, so that the distance was larger for seat #1 than for seat #2. This was achieved by increasing the size of the table at which seat #1 was located.

For the *“anchor”* scenario (Figure 3), the amount of privacy was varied by placing one table, with seat #1, close to the wall (‘anchored’ table) and letting the other table, with seat #2, be surrounded by other tables containing people (‘unanchored’ table).

#### Measures

##### Seat choice

In each scenario, participants indicated which of two seats they preferred. Seat choice was measured using a five-point Likert scale, in which 1 = “definitely seat 1,” 2 = “probably seat 1,” 3 = “no preference,” 4 = “probably seat 2,” and 5 = “definitely seat 2.” Scores were recoded so that higher scores reflect a choice for the low-intimacy or high-privacy seat. From the resulting scores we detracted 3 in order to center the means at zero. For the choice scores so created, positive scores reflect a choice for the low-intimacy (Scenario 1 and 2) or high-privacy seat (Scenario 3), which would be in line with our hypotheses, while negative scores reflect a choice for the high-intimacy/low-privacy seat, and a score of zero reflects no preference for either seat.

##### Manipulation checks

For every scenario participants had to indicate whether they agreed or disagreed with a statement describing the manipulation that was performed for that particular scenario. For the eye contact scenario the statement read: *“When I take seat 1, I will be sitting directly opposite another café-goer; when I take seat 2 I will be sitting not directly opposite another café-goer”*; and for both the distance scenario and the anchor scenario the statement read: *“When I take seat 1, I will be sitting further away from the other café-goers; when I take seat 2, I will be sitting closer to other café-goers.”*

##### Procedure

Participants were asked by the instructor whether they wanted to partake in a study about social interaction in a café environment. Questionnaires were distributed in a classroom, after which participants were given time to complete them. The consent procedure was followed as described in the earlier paragraph. Then the first page of the questionnaire explained the setting of the experiment, and it was stressed that it would be important for the participants to imagine themselves to be in a real café during the experiment. On the next page, participants read about the café atmosphere for the first scenario and saw a floor plan depicting an imaginary café, after which they completed the dependent measures for that scenario. After the first scenario there followed the second and the third scenario, each accompanied by their own set of dependent measures and each preceded by a description of the café atmosphere.

After completion of the items for the third scenario participants were asked to complete the items used as checks, and to indicate their age and gender. After all questionnaires had been filled in, they were collected, and participants were thanked and debriefed.

### Results

#### Checks

In Scenario 1, 92% of participants correctly indicated that there was a difference between seat 1 and seat 2 in terms of gaze direction of the other café-goer. In Scenario 2, 88% of the participants indicated there was a difference between both seats in terms of spatial proximity to other café-goers. This percentage was lower for Scenario 3 (75%).^2^

Since participants did correctly answer manipulation checks 1 and 2 (92% and 88% correct, respectively), there was no reason to assume that participants were unable to interpret correctly the spatial layout of the simulated café.

#### Seat Choice

When asked to indicate their choice for either of the vacant seats, most participants indicated a choice for one of the two seats over the “no preference” option (see Table 1). We hypothesized that participants would choose a seat that did not facilitate eye contact over a seat that did facilitate eye contact with another café-goer. This hypothesis (H1a) was, however, not supported by the data: although the seat choice scores in Scenario 1 did suggest a tendency to choose the low eye contact seat (*M* = 0.20, *SD* = 1.26), it did not differ significantly from the *no preference* option (0), *t*(70) = 1.32, *p* = 0.192.

**Table 1:** Seat choice per scenario.

	Scenario 1: Eye-contact		Scenario 2: Distance		Scenario 3: Anchoring	
	Choice (*N*)	Choice %	Choice (*N*)	Choice %	Choice (*N*)	Choice %
Surely seat 1	8	11	19	27	15	21
Probably seat 1	31	44	24	34	19	27
No preference	8	11	1	1	6	9
Probably seat 2	15	21	17	24	23	32
Surely seat 2	9	13	9	13	7	10
Total	71	100%	70	100%	70	100%

*Original items were in Dutch.*

*Seat 1: low intimacy/high privacy. Seat 2: high intimacy/low privacy (Study 1)*.

Support for hypothesis 1b was found: participants indicated to prefer the far seat (*M* = 0.39, *SD* = 1.44) over the seat in close proximity to other café-goers at the same table, *t*(69) = 2.25, *p* = 0.028. Hypothesis 1c was not supported: although scores suggested a preference for the anchored seat (*M* = 0.17, *SD* = 1.36), this effect was not significant, *t*(69) = 1.05, *p* = 0.296.

### Discussion

We hypothesized that participants who had to imagine being in a café alone would feel discomfort having to sit in close proximity to café-goers that were strangers to them. We based this thought on the finding that inhabitants of Western society reserve the area immediately surrounding them, up to 1.2 m (4 ft.), for intimate and personal social transactions (Hall, 1968), and that being seated at a café table where a stranger was already seated would mean infringement on both the participant’s space and that of the other. Although Hall’s estimates of space zones are general, and certainly not tailored to the café context, we hypothesized that participants might judge the situation to be too intimate, and prefer seats that provide lower intimacy—in accordance with affiliative conflict theory (Argyle and Dean, 1965)—or more privacy—in accordance with privacy regulation theory (Altman, 1975).

Only one of three hypotheses was supported by the data: participants preferred a seat that was at a larger distance to three other café-goers seated at the same table (H1b). They were indifferent about direction of gaze (H1a) and anchoring (H1c). It is notable, however, that the non-significant effects were in the expected direction, suggesting a possible inclination for the low intimacy and the high privacy seats.

While this first study provided some preliminary evidence for the effect of intimacy on seating behavior, it remains ambiguous to which deeper concerns these effects relate. Study 2 attempts to replicate the findings of Study 1, using a larger sample of participants, in order to increase the reliability of effects. In addition, Study 2 examines affective and socio-cognitive attributes of seat choice in the high-intimacy and low-privacy situations we devised.

## Study 2

Study 2 is a replication of Study 1. Added were measures of affect, derived from the circumplex model of affect (Russell, 1980; Yik et al., 2011), and of cognitive appraisals pertaining to the different seats. The circumplex model of affect (Russell, 1980) distinguishes two dimensions of affect: valence (pleasure) and arousal. Although pleasure is thought to be important, so is arousal because different degrees of arousal, with the same degree of pleasure, define distinctly different emotions (Russell, 1980). We predicted that seat choice would be related to both the pleasure and the arousal dimension of affect. Specifically, we expected a choice for the seat that was experienced as more pleasant (Hypothesis 2a) and less arousing (Hypothesis 2b; Middlemist et al., 1976; Coutts et al., 1980; Evans and Wener, 2007).

Not much is known about the cognitions preceding or underlying proxemic behavior in specific contexts. Recently, Staats and Van der Jagt (unpublished) discerned 16 cognitive appraisals, specific to seat choice in a café. That study looked at a café setting in which there was a choice to seat oneself at a small table or at a reading table, in all cases with other people – strangers – present at the same table. For that study the set of relevant beliefs was created in qualitative exploratory research, in the tradition of Fishbein and Ajzen (2010; or see Ajzen’s (2018) website^3^) of collecting salient beliefs. The selection of 8 items made for this study was based on the specific context of this study and the statistical quality of a number of the items in the other study (see items in Table 3). It was intended to explore whether and how context-specific cognitions could provide insight into the decision where to sit in a café. The items bear a resemblance to the items used by Robson et al. (2011, Exhibit 2, p. 256) who investigated privacy concerns in a restaurant setting.

### Methods

#### Participants, Consent Procedure, and Design

Participants were 121 pre-university students attending an introductory psychology lecture at a university in the Netherlands (84% female, mean age = 17.1 years). As in Study 1, participants were asked to participate voluntarily in the current experiment, and following the exact same procedure. All participants received the same questionnaire, which contained three different café scenarios that were presented in a fixed order.

#### Scenarios, Manipulations, Procedure, and Measures

These were identical to Study 1 apart from the following: In addition to recording participants’ preferred seats, participants had to rate *both* seats—the one they chose and the one they rejected—for their affective and cognitive properties. For each scenario participants completed a set of items based on the following concepts:

##### Affective appraisals

Five items were used to measure the pleasure and arousal that being seated at either of the two vacant seats would elicit in the participants: ‘pleasurable,’ ‘distressing,’ ‘relaxed,’ ‘boring,’ and ‘exciting.’ Answer scales ranged from 1 (“definitely not”) to 5 (“definitely”). Answers were recorded separately for both vacant seats, so that in total ten ratings of affect were obtained per scenario.

##### Cognitive appraisals

Eight items were used to measure the cognitive appraisals that being seated at either of the two vacant chairs would elicit in the participants. The text “In seat [1 or 2] I think that…” was followed by eight items measuring different cognitions (e.g., “people will think I am lonely”; “I can do something for myself without being disturbed,” see Table 4). Participants had to respond to eight items per vacant seat, so that in total 16 ratings were obtained per scenario.

##### Seat choice

As in Study 1, seat choice was measured using a five-point Likert scale, in which 1 = “definitely seat 1,” 2 = “probably seat 1,” 3 = “no preference,” 4 = “probably seat 2,” and 5 = “definitely seat 2.” Scores were recoded so that higher scores reflect a choice for the low-intimacy (Scenario 1 and 2) or high-privacy (Scenario 3) seat. From the resulting scores we detracted 3 in order to center the means at zero. For the choice scores so created, positive scores reflect a choice for the low-intimacy/high-privacy seat (in line with our hypotheses), while negative scores reflect a choice for the high-intimacy/low-privacy seat, and a score of zero reflects no preference for either seat.

##### Manipulation checks

These were the same as used in Study 1.

#### Statistical Analysis

In order to examine the affective and cognitive characteristics attributed to the chosen and to the non-chosen seat per scenario, we split the participants in two groups for each scenario. Groups consisted of people who chose the same seat. Differences between their scores of the seat they chose compared to their scores of the non-chosen seat were statistically tested with *t*-tests. A second step in the analysis was to use these difference scores in tests (*t*-tests again) to see whether these differed in extremeness. It could for example be the case that degree of pleasure attributed to one seat by the group who chose this seat was more extreme than the degree of pleasure attributed to the other seat by the other group who chose the other seat. In such a case the choice of the first group is more outspoken regarding pleasure. We did this for all affective and cognitive characteristics.

### Results

#### Preliminary Analyses

##### Missing value analysis

The total amount of missing values was quite low (<1% for all questions except seat choice in the privacy condition [1.7%] and the manipulation check in that condition [5%]).

##### Manipulation checks

Participants correctly answered Manipulation Checks 1 and 2 (95 and 97% correct, respectively), indicating that they correctly perceived that, in the eye contact scenario, one seat offered more eye contact than the other, and that, in the distance scenario, one seat was closer to other people at the same table than the other. However, due to using the same ambiguously interpretable manipulation check as in Study 1, only 69% of the participants correctly answered Manipulation Check 3, which was used in the anchoring scenario. Again, since participants did correctly answer manipulation checks 1 and 2 there was no reason to assume that participants were unable to interpret correctly the spatial composition of the simulated café (see also Note 2).

##### Component analyses

Data analysis first required reducing the five affective appraisal items and eight cognitive appraisal items to a smaller number of interpretable dimensions. To that end, principal component analysis (PCA) was conducted with the five affective appraisal items and again with the eight cognitive appraisal items.

We first summed scores for each of the affective appraisal and cognitive appraisal items across the two seats and three scenarios in order to obtain mean affective appraisal and mean cognitive appraisal scores that were then used for scale construction. This procedure has previously been used for example by Staats et al. (1997). The mean scores so created reflected participants’ affective and cognitive responses to seats in a café, irrespective of the seat that was judged or the experimental scenario that was used. For these summed-across-conditions variables, the PCA analysis resulted in a two-factor solution for the affective appraisals and in a three-factor solution for the cognitive appraisals.

For the affective appraisal variables, a factor structure emerged that was in line with the circumplex model of affect (Russell, 1980). The first factor reflected a general sense of pleasure, and the second factor reflected arousal (Table 2).

**Table 2:** Rotated component loadings for affective appraisal appraisals of seats in a café setting (Study 2).

Items	Pleasure	Arousal
Pleasurable	0.84	–0.22
Distressing	–0.34	0.82
Relaxed	0.67	–0.51
Boring	–0.64	–0.24
Exciting	0.18	0.88
Eigenvalue	1.26	2.24
% Variance explained	25.2	44.9

*Original items were in Dutch*.

For the cognitive appraisal variables, a factor structure emerged that uncovered three components which we labeled “lonely/pathetic,” “voluntary contact,” and “obliged contact,” with the distinction between voluntary and obliged contact being that voluntary contact conveys a sense of positive contact seeking by the participant, whereas obliged contact conveys a sense of having to respond (unwillingly) to other people’s attempt at contact (Table 3). The “I will disturb the others’ privacy” item cross-loaded on the *lonely/pathetic* (0.64) and *obliged contact* component (0.46). We couldn’t interpret how this item could be part of either component, and therefore decided to not include the item in any component.

**Table 3:** Rotated component loadings for cognitive appraisal of seats in a café (Study 2).

Items	Lonely/pathetic	Voluntary contact	Obliged contact
People will think I’m pathetic.	0.90	–0.05	–0.04
People will think I’m lonely.	0.94	–0.04	0.02
Others won’t make contact with me.	0.30	–0.56	0.17
I can do something for myself without being disturbed.	0.12	0.11	–0.86
I can easily make contact with other nice people.	0.07	0.88	0.17
I will disrupt others’ privacy.	0.64	0.02	0.46
I can regulate contact with others well.	0.04	0.85	0.04
Others will expect me to start a conversation.	0.37	0.33	0.73
Eigenvalue	2.60	1.99	1.24
% Variance explained	32.4	24.9	15.4

*Original items were in Dutch*.

For all subsequent analyses, we created scales by multiplying the individuals’ item ratings per seat in each scenario by the factor loadings found for the summed-across items (Tables 2, 3), and then adding them. All subsequent analyses used the factor scores so created for each individual in each scenario. When the reliability of these scales was tested across the three scenarios (i.e., items from all three scenarios were included in the analysis simultaneously), alphas for these scales indicated acceptable (α_arousal_ = 0.780; α_voluntary_contact_ = 0.724; α_obliged_contact_ = 0.785) to good (α_pleasure_ = 0.839; α_lonely/pathetic_ = 0.876) internal consistency.

#### Main Analyses

##### Seat choice

When asked to indicate their choice for either of the vacant seats, most participants indicated choice for one of the two seats over the “no preference” option. In the eye contact scenario 15 out of 120 participants indicated no preference, in the distance scenario this was 6 out of 120, and in the anchoring scenario 19 out of 119 (Table 4).

**Table 4:** Seat choice per scenario.

	Scenario 1: Eye-contact		Scenario 2: Distance		Scenario 3: Anchoring	
	Choice (*N*)	Choice %	Choice (*N*)	Choice %	Choice (*N*)	Choice %
Surely seat 1	10	8	25	21	17	14
Probably seat 1	72	60	34	28	44	37
No preference	15	13	6	5	19	16
Probably seat 2	23	19	36	30	33	28
Surely seat 2	0	0	19	16	6	5
	120	100%	120	100%	119	100%

*Original items were in Dutch.*

*Seat 1: low intimacy/high privacy. Seat 2: high intimacy/low privacy (Study 2)*.

According to Hypothesis 1a, participants would choose a low eye contact seat, which restrains intimacy, over a high eye contact seat, which promotes intimacy. In support of this hypothesis, most participants opted to take the low eye contact seat (*N* = 82) over the high eye contact seat, (*N* = 23). Choice for the low eye contact seat was significantly above the scale midpoint (“no preference”), *M* = 0.57, *SD* = 0.90, *t*(119) = 7.0, *p* < 0.001^4^.

Hypothesis 1b, which states that participants would choose the seat that was farthest away from others at the same table, and thus being the least intimate seat, was not confirmed. Participants did not choose the high distance-to-others-at-same-table seat (*N* = 59) over the low-distance-to-others seat (*N* = 55) in Scenario 2, *M* = 0.08, *SD* = 1.44, *t*(119) = 0.64, *p* = 0.263.

According to Hypothesis 1c, participants would prefer an anchored table, facilitating privacy, over a non-anchored table. Support for this hypothesis was found as participants made a choice for the anchored seat (*N* = 61) over the non-anchored seat (*N* = 39). Choice for the anchored seat was therefore significantly above the scale midpoint, *M* = 0.28, *SD* = 1.17, *t*(118) = 2.6, *p* = 0.006.

##### Hypothesis tests for pleasure and arousal

According to Hypotheses 2a and 2b seat choice would be positively related to pleasure ratings of a seat, and negatively related to arousal ratings. Table 5 displays how participants rated pleasure and arousal for their seat of choice, compared to the non-chosen seat. Each number represents a within-participants difference score that reflects how much higher or lower participants rated their seat of choice compared to the non-chosen seat on a given appraisal dimension, a relative score. A relative score is *italicized* when it significantly different (*p* < 0.05) from zero. Zero implies that there is no difference between the chosen and the non-chosen seat on that dimension. Relative scores are calculated for the two groups that chose one of the two seats in each scenario. When the *superscripts* of a pair of relative scores are different this signifies that the relative scores of the seats chosen by the two groups differ significantly (*p* < 0.05).

**Table 5:** Mean relative scores (score of chosen seat minus non-chosen seat) per chosen seat per scenario (Study 2).

Scenario	Eye contact		Distance		Anchoring	
Chosen seat	Low intimacy	High intimacy	Low intimacy	High intimacy	High privacy	Low privacy
*N* participants who chose this seat	82	23	59	55	61	39
Pleasure	*1.35^a^*	*0.79^a^*	*1.00^c^*	*2.64^d^*	*1.35^e^*	*0.96^e^*
Arousal	*–2.04^a^*	*–*0.21^b^	*–3.22^c^*	*1.50^d^*	*–2.00^e^*	0.41^f^
Sad-lonely	0.29^a^	*–0.76^b^*	0.40^c^	*–2.35^d^*	0.18^e^	*–0.92^f^*
Voluntary contact	0.01^a^	*–*0.07^a^	*–2.21^c^*	*3.70^d^*	*–0.44^e^*	*1.70^f^*
Obliged contact	*–1.49^a^*	0.02^b^	*–3.31^c^*	*2.91^d^*	*–0.67^e^*	*0.60^f^*

*Italicized means represent within-participant relative scores that differ from zero at p < 0.05. Means with different superscripts within the same scenario represent relative scores that differ between groups*.

Being able to analyze the affective and cognitive appraisals of each of the seats means the tests of hypotheses 2a and 2b are different from the hypothesis tests for hypotheses 1a, 1b, and 1c. Each choice of seat can be investigated for its ratings on pleasure and arousal as well as its cognitive characteristics, both the one chosen most often (in the eye contact and the anchor scenario as predicted, not in the distance scenario), as the other, less often chosen seat. We tested hypotheses for pleasure and arousal for the low intimacy/high privacy seat in each scenario in the spirit of the two theories.

The relative scores in Table 5 provide support for Hypotheses 2a and 2b in all three scenarios. Participants who chose the low intimacy or high privacy seat rated these as more pleasurable and less arousing than the rejected seat. This shows in the eye contact scenario with a relative pleasure score (*M* = 1.35) and a relative arousal score (*M* = -2.04), indicating that pleasure was considered higher and arousal lower than for the non-chosen seat. It is also true for the distance scenario: more pleasure (*M* = 1.00), and lower arousal (*M* = -3.22), expected for the low intimacy (but not chosen significantly more often) relative to the high intimacy seat. And it is true for the anchor scenario: more pleasure (*M* = 1.35), and less arousal (*M* = -2.00) for the high privacy seat.

##### Affective/cognitive profiles of seats

However, the information obtained allows more to be learned from the participants’ ratings and that easily shows from the information in Table 5: by having information about both seats, provided by everyone, we know how *both* groups rate *each* chair. Test of the hypotheses needed only to regard the characteristics of the choice expected by the theories. However, the information we have allows a deeper understanding of the choices made as we also know how choice for the high intimacy seats in Scenario 1 and 2 and the low privacy seat in Scenario 3 can be understood. In the *eye contact* scenario not only participants who chose the low intimacy seat (i.e., the low eye contact seat) but also participants who chose the high intimacy seat (i.e., the high eye contact seat) indicated that they found their own seat more pleasurable than the other, as is indicated by the positive (italicized) mean relative scores for pleasure in Table 5. The identical superscripts mean that both groups expect the same degree of pleasure from their choice relative to the non-chosen seat. Regarding arousal participants who had chosen the low-intimacy seat rated that seat as significantly less arousing than the high-intimacy seat, whereas participants who had chosen the high-intimacy seat did not perceive a difference between the seats, implying that for them arousal was not a criterion for choice. The difference between these relative scores was significant for the arousal variable, as shown in the different superscripts.

The three cognitive dimensions allow an interpretation of these affective profiles. Participants who chose the low intimacy seat (i.e., the low eye contact seat) indicated that they did not expect to be perceived as more or less sad/lonely at their seat than at the other seat, whereas participants who chose the high intimacy seat (i.e., the high eye contact seat) indicated that they expected this stronger exposure would make them being perceived less sad or lonely than in the non-chosen (low eye contact) seat, as indicated by the negative relative score for sadness/loneliness. The difference between these relative scores was significant (different superscripts in Table 5 in the eye contact panel), suggesting that for the group choosing the high intimacy seat this opposite of being considered sad or lonely contributed to the choice. Regarding the opportunity for voluntary contact, both participants choosing the low-intimacy seat and those choosing the high-intimacy seat indicated that they perceived no difference between the two seats. Lastly, participants choosing the low-intimacy seat indicated that they expected less obliged contact at that seat than at the other, whereas participants choosing the high-intimacy seat did not perceive a difference. The different superscripts show that this dimension was relevant for choice only for the group choosing the low intimacy seat.

In the *distance* scenario more pronounced differences emerged. Again, both groups rated their own seat as significantly more pleasurable than the seat that they had rejected. However, participants who had chosen the high-intimacy seat (small distance to others) did so more extremely than participants who had chosen the low-intimacy seat (mean difference scores of 2.64 vs. 1.00, significant as shown in the different superscripts). Clearly, they expect more pleasure from their choice than the ones choosing the low intimacy seat. Furthermore, participants who had chosen the low-intimacy seat rated that seat as significantly less arousing than the high-intimacy seat, while participants who had chosen the high-intimacy seat rated their seat as significantly *more* arousing than the low-intimacy seat (mean relative scores of -3.22 vs. 1.50; significant difference shown in the different superscripts). Scores on the three cognitive dimensions were also all different: Participants who opted for the low-intimacy seat generally expected the same sadness/loneliness at their seat as at the other seat (non-significant relative score) while participants who opted for the high-intimacy seat on the other hand expected to be perceived as less sad/lonely at their seat than at the other seat. The difference between these relative scores was significant (different superscripts). Participants who opted for the low-intimacy seat indicated that they saw less opportunity for voluntary contact with another café-goer at that seat, while participants choosing the high-intimacy/low-privacy seat indicated that they saw much more opportunity for voluntary contact at their seat (all *p*s < 0.05). The difference between these relative scores was significant. Finally, participant choosing the low-intimacy seat also expected to have to oblige less to others’ attempts at contact at their seat than at the other seat, whereas participants opting for the high-intimacy seat expected more obliged contact at their seat than at the other. Again, the difference between these relative scores was significant.

In the *anchoring* scenario both groups again rated the seat they chose as more pleasurable than the rejected seat and to the same degree, the difference between their scores not being significant. Participants choosing the high-privacy seat regarded that seat as less arousing than the low privacy seat; participants who chose the low-privacy seat did not rate their seat as more or less arousing than the other seat. The difference between these relative scores was significant for the arousal variable. All three cognitive dimensions displayed differences between the groups: the group choosing the high privacy seat did not expect differences regarding being perceived as sad or lonely, but the low privacy group expected a positive outcome: a better impression than in the other seat. Voluntary contact and obliged contact followed the same pattern in this scenario: less contact expected for those in the high privacy seat, more contact expected in the low privacy seat.

### Discussion

In two out of three scenarios, participants chose one over the other seat in line with predictions. In the eye contact scenario participants indicated choice for the low eye contact seat (low intimacy), and in the anchoring scenario participants indicated choice for the anchored seat (high privacy). These findings support Hypotheses 1a and 1c, which stated that individuals who have to choose between two seats in an otherwise completely occupied café would prefer seats of low intimacy and of high privacy, being seats that restrain eye contact and seats that are located next to a wall. No support was found for Hypothesis 1b, which stated that participants would choose a seat that was at a larger distance from others at the same table; participants were equivocal.

In this second study we also set out to investigate the relationships between seat choice and affective and cognitive appraisals of the available seats. Analyses of relative scores on these appraisals revealed many differences between participants choosing the low-intimacy/high-privacy seat and participants choosing the high-intimacy/low-privacy seat. Generally, participants choosing the low-intimacy/high-privacy seat regarded their seat as less arousing and more pleasurable than participants choosing the high-intimacy/low-privacy seat, and were neutral about being regarded as sad or lonely there, compared to the other seat. This is really different for the participants choosing the high intimacy/low privacy seat. They seem to perceive this choice as creating a better image than in the other seat, having negative relative scores on the sad/lonely dimension. The three scenarios also provide fairly consistent differences on the two contact dimensions: negative relative scores for the low intimacy/high privacy seats, showing that choice for these seats is based on the expectation that one can be comfortably alone. The groups choosing the high intimacy/low privacy seats, however, appear to look forward to contact, having positive scores for the voluntary as well as the obliged contact dimensions. It is just the voluntary contact dimension for the eye contact scenario that has neutral scores for both seats, showing that this is apparently no issue for this scenario, while obliged contact for this scenario only shows a negative relative score for the low intimacy seat, seemingly a better place to avoid this.

## General Discussion

In two studies we investigated whether seat choice of café-goers is determined by expectations of intimacy or privacy. The imaginary café in our experiments was presented as a crowded place in which people were seated in close distance to each other. This close proximity of unfamiliar others was designed to present a potential threat to the personal space integrity of the café-goer (Hall, 1966), and we hypothesized that they would respond by choosing a seat that allowed them to create psychological distance. In both studies we applied the same three scenarios to manipulate intimacy and privacy, two distinct concepts of interpersonal space, as we interpreted it. In two scenarios we tested how the intimacy of the setting would influence seat choice, the third scenario was devised to investigate privacy. We conceptualized intimacy as psychological closeness resulting from interaction with another person, and derived two manipulations from this concept. We found that participants were sensitive both to the amount of possible eye contact with another café-goer (in Study 2, not in Study 1), and to the physical distance to others at the same table (in Study 1, not in Study 2). Where confirmed these findings are in line with affiliative conflict theory, which predicts that individuals seek to maintain a balance between intimacy and personal freedom (Argyle and Dean, 1965). In a third scenario we tested whether privacy affected participants’ seat choice. We conceptualized privacy as a one-sided mechanism allowing people to have “selective control of access to the self” (Altman, 1975, p. 18), and manipulated the amount of available privacy by placing one empty seat at a table close to a wall and another at a table in the middle of the café. We found that participants were sensitive to this manipulation and overall preferred the high-privacy seat (in Study 2, not in Study 1). Since in the intimacy scenarios the amount of privacy between seats was held constant, and in the privacy scenario the amount of intimacy between seats was held constant, we may conclude that intimacy and privacy concerns both have a unique predictive impact on seat choice in cafés.^5^

But this was definitely not all. Maybe the most interesting findings came from the outcomes *not* predicted by these theories. After all, not all hypotheses were confirmed in each study, and confirmation was not overly strong either, suggesting that other motives also influenced the outcomes. Due to the methodology chosen for the analysis of affective and cognitive characteristics that we collected ratings on in Study 2, a much more detailed picture appeared. That picture suggests a split between groups: one group choosing conservatively, looking for a calm place where other people present – strangers – would not oblige them to communicate and where self-initiated conversation was also not likely. This is in line with previous research demonstrating the positive relationship between arousal and unwanted intimacy (Middlemist et al., 1976; Coutts et al., 1980) or the loss of privacy (Webb, 1978). But then there was the other group, in most scenarios across the two studies not so much smaller, choosing a seat that they expected to provoke excitement, and where communication with the other persons, obliged or self-initiated, was more likely. Further research could benefit from the use of personality measures dealing with introversion-extraversion or more particular measures on social anxiety (e.g., Liebowitz, 1987). Another potential avenue of further explanation might focus on normative considerations as to what is appropriate in a café with regard to contacts with strangers. Some of the items measuring cognitions focused on normative expectations but this phenomenon could be researched more thoroughly.

We obtained these findings using a very modest way of representing the environment settings: simple floor plans on a sheet of paper accompanied by a minimal description of the atmosphere and the activity of the participant. This can be considered a weakness of the method, as choices in high density situations are known to be influenced by activity, gender, and age differences^6^. Nevertheless the results are not weak, but show many explicit and plausible differences between the choices made, suggesting that the situation evokes fairly strong ideas about what will be experienced and how to behave with regard to those anonymous strangers at the table. The current results are obtained with young samples of participants who may differ from other age groups in their sociability. We don’t know if this is in fact the case, as to the best of our knowledge a similar methodology to analyze seating choices in public settings has not been done. We think the method might be fruitfully expanded to situations where immersion is higher, e.g., in virtual environments or of course in real settings, with other age groups, and more elaborately described activities. Other settings, like waiting rooms or libraries, would also be interesting to compare with the café which seems, after all, to be a setting that, at least for a large minority, evokes sociability tendencies. Advantage of the current method is its simplicity which makes it easy to be used for applied purposes.^7^ Validity of the method may need further proof but the current results suggest that powerful results can be obtained.

Important to address is that all manipulations developed and tested in this paper are ultimately based on the premise that social contact in the personal zone, i.e., within 1.2 m of the subject, should be restricted to intimates and friends, and not to strangers. Obviously this goes back to Halls’s distinction of zones. It was the overall research question of this paper to see how privacy and intimacy mechanisms play out in this zone, in a café setting. One might argue that Hall’s distinction of zones is general and not tailored to the café situation in a western country like the Netherlands. There is some cross-cultural work done, one study in particular that looked at interpersonal distances in 42 countries, that suggests fairly strong agreement among countries as to the acceptable distance to strangers (Sorokowska et al., 2017, in particular Figure 2). These findings do not pertain to café environments but the distance to strangers that we suggest in the scenarios, between 70 and 100 cm, is a distance that is smaller than desirable in most of the countries involved in the study. We did not have more specific information to go on. However, it seems that participants were rather sensitive to the distinctions we made. We conclude that at least as a rule of thumb a distance measure in the spirit of Hall’s theory proved useful. Regarding the age of the two theories involved and possible modifications in more recent years we have seen that the amount of research dedicated to each theory seems to have diminished somewhat but is still substantive. More recent work shows that the core of the theories has not really changed (e.g., Westin’s, 2003 paper in the Journal of Social Issues), although it has given rise to more specific theories, like Communication Privacy Management (CPM, in Margulis, 2003). Hall’s model for interpersonal distances has also remained pretty much intact, as for example reported in Layden et al. (2018) (PLoS ONE, p. 4) and the paper by Sorokowska et al. (2017), already referred to. We think that our manipulations remained close to the original core of the theories, and that their value for us was to get to grips with an everyday phenomenon, and understand it better. In that sense our theoretical aspirations were modest.

The implications of our findings for café-owners are not straightforward for a number of reasons. First of all this study was designed to test a number of hypotheses stemming from two theories on proxemics. The scenario’s developed to test these theories hardly compromised to create a situation that resembled any specific café in a realistic context. This led to manipulations creating conditions that are rare: Being alone among strangers in a café where only two seats are still available and nevertheless deciding to stay there is not a very common experience. Of course this was necessary to create results that were interpretable from our theoretical expectations. In addition we employed a population of rather young participants, with preferences that might differ from other populations that frequent cafés. We should add though that the young participants were familiar with cafés and had no difficulties imagining the situation (see Footnote 6). We also did not refer to familiarity with the café, or the possibility to find groups of like-minded visitors, and many other social factors that will affect choices, mood and the pleasure of visiting. In that sense the study mainly contributes on a rather general level to insights that might help in creating a pleasant ambiance. Nevertheless, we think it is questionable how aware café owners are of psychological consequences of the design of their café in relation to visitor pressure. So we restrict suggestions for application of our findings to the advice that it may be helpful for café owners to be sensitive to intimacy and privacy issues, that identifiable features of the interior influence these experiences, that visitors’ preferences may differ strongly, and that pleasure, repeat visits, and ultimately the profit made in the café, will partly depend on how these issues are dealt with.

## Author Contributions

HS developed and ran the experiments and partially wrote the manuscript. PG analyzed the data and wrote parts of the manuscript.

## Conflict of Interest Statement

The authors declare that the research was conducted in the absence of any commercial or financial relationships that could be construed as a potential conflict of interest.

## Footnotes

1 This definition seems not completely logical: privacy is not a state that can be overdone. Complete privacy assumes perfect control, which also may imply the desire to communicate with others, individually or in a group, provided that the subject maintains control. However, in the present study, in particular the scenario based on privacy, this discrepancy is not a problem.

2 An explanation for this relatively low success rate for the third manipulation check might be that the manipulation check itself was poorly formulated. In the anchoring scenario, participants were asked to answer the following question: *“When I take seat 1, I will be sitting further away from the other café-goers; when I take seat 2, I will be sitting closer to other café-goers.”* This question was used to check whether participants correctly perceived that the high privacy seat (which was located close to a wall) was more distant to possible sources of privacy intrusion—that is, people seated at nearby tables—then the low privacy seat. Exactly the same check had, however, been used in the preceding distance scenario, where it was used to check whether participants correctly perceived that they were more distant to other participants seated at the same table. It is plausible that participants’ attention was therefore drawn to the position of other persons seated at the same table, rather than persons seated at other tables. And since in the anchoring scenario the two different seats were at tables of exactly the same size, participants might have thought that there was no difference in distance to other persons in the anchoring scenario.

3 http://people.umass.edu/aizen/pdf/tpb.measurement.pdf

4 Given that seat choice did not meet the normal distribution criterion necessary for *t*-tests, we also performed Wilcoxon signed rank tests which do not require normal distribution. The results from these Wilcoxon signed rank tests did not differ from those obtained with the *t*-tests, and therefore we chose to display the original results.

5 We followed the suggestion of one of the reviewers to pool the data, given that the two samples were so similar, although data collection for the two studies was a year apart. Results were that the effects increased in strength; for eye contact *t*(190) = 5.673, *p* = 0.000; for distance at the same table *t*(190) = 1.864, *p* = 0.064; for anchoring *t*(190) = 2.643, *p* = 0.009. Thus aggregated, the effects are stronger and even the ‘distance’ manipulation is marginally significant overall. In itself this is not so surprising given that the separate studies already had significant effects or showed tendencies in the expected direction.

6 We did not observe effect of gender: we analyzed the data of study 2 for women only. Results were virtually identical for the three hypotheses: for eye contact *M*_womenonly_ = 0.56 while this was *M* = 0.57, for the whole sample. For distance *M*_womenonly_ = 0.10, while *M* = 0.08 for the whole sample. For anchoring *M*_womenonly_ = 0.27 while *M* = 0.28 for the whole sample. Excluding the 20 males did not change the outcomes, suggesting that gender did not clearly affect the results.

7 We have additional data, not reported in the main text, regarding the two samples’ experience with cafés and their evaluation of the simulation material. At the end of the questionnaire of the first and the second study we asked about their visits to cafés (for at least 1 h): whether they liked going, how often they had visited a café during the last 3 months, how good the floor plans captured the impression of a café and whether they considered the questions clear. Our participants apparently had visited cafés with an average of approximately 7 times during the last 3 months (both studies), they liked going there (*M*_Study__1_ = 3.46, *M*_Study__2_ = 3.44, on a 5-point scale), they considered the floor plans to give a reasonably good impression (*M*_Study__1_ = 2.90, *M*_Study__2_ = 2.98, both on a 5-point scale), and found the questions clear (*M*_Study__1_ = 5.63 on a 7-point scale, *M*_Study__2_ = 3.75 on a 5-point scale). We think these scores allow the conclusion that our participants were able to give valid judgments of the scenario’s.
